# Multi-omics integration to identify the genetic expression and protein signature of dilated and ischemic cardiomyopathy

**DOI:** 10.3389/fcvm.2023.1115623

**Published:** 2023-02-13

**Authors:** Konstantina Portokallidou, Nikolas Dovrolis, Georgia Ragia, Natalia Atzemian, George Kolios, Vangelis G. Manolopoulos

**Affiliations:** ^1^Laboratory of Pharmacology, Medical School, Democritus University of Thrace, Alexandroupolis, Greece; ^2^Individualised Medicine and Pharmacological Research Solutions Center, Alexandroupolis, Greece; ^3^Clinical Pharmacology Unit, Academic General Hospital of Alexandroupolis, Alexandroupolis, Greece

**Keywords:** heart failure, dilated cardiomyopathy, ischemic cardiomyopathy, precision medicine, proteomics, transcriptomics, omics integration

## Abstract

**Introduction:**

Heart failure (HF) is a complex clinical syndrome leading to high morbidity. In this study, we aimed to identify the gene expression and protein signature of HF main causes, namely dilated cardiomyopathy (DCM) and ischemic cardiomyopathy (ICM).

**Methods:**

Omics data were accessed through GEO repository for transcriptomic and PRIDE repository for proteomic datasets. Sets of differentially expressed genes and proteins comprising DCM (DiSig) and ICM (IsSig) signatures were analyzed by a multilayered bioinformatics approach. Enrichment analysis *via* the Gene Ontology was performed through the Metascape platform to explore biological pathways. Protein-protein interaction networks were analyzed *via* STRING db and Network Analyst.

**Results:**

Intersection of transcriptomic and proteomic analysis showed 10 differentially expressed genes/proteins in DiSig (*AEBP1*, *CA3*, *HBA2*, *HBB*, *HSPA2*, *MYH6*, *SERPINA3*, *SOD3*, *THBS4*, *UCHL1*) and 15 differentially expressed genes/proteins in IsSig (*AEBP1*, *APOA1*, *BGN*, *CA3*, *CFH*, *COL14A1*, *HBA2*, *HBB*, *HSPA2*, *LTBP2*, *LUM*, *MFAP4*, *SOD3*, *THBS4*, *UCHL1*). Common and distinct biological pathways between DiSig and IsSig were retrieved, allowing for their molecular characterization. Extracellular matrix organization, cellular response to stress and transforming growth factor-beta were common between two subphenotypes. Muscle tissue development was dysregulated solely in DiSig, while immune cells activation and migration in IsSig.

**Discussion:**

Our bioinformatics approach sheds light on the molecular background of HF etiopathology showing molecular similarities as well as distinct expression differences between DCM and ICM. DiSig and IsSig encompass an array of “cross-validated” genes at both transcriptomic and proteomic level, which can serve as novel pharmacological targets and possible diagnostic biomarkers.

## 1. Introduction

Despite contemporary advances in medicine, cardiovascular diseases (CVDs) are still the leading cause of mortality worldwide, accounting for almost half the total number of global deaths (1). CVDs encompass a wide array of heart and vessel-related pathologies, such as coronary heart disease, hypertension, cardiomyopathy (CM), and congenital heart disease (2, 3), all eventually progressing to heart failure (HF).

Heart failure is a debilitating condition that manifests as a consequence of abnormalities in cardiac function, structure, rhythm, or conduction (4). The etiological factors of HF syndrome are often difficult to discern and vary (5, 6), with dilated cardiomyopathy (DCM) and ischemic cardiomyopathy (ICM) being among the main causes of HF in Western countries. ICM is defined by an imbalance between myocardial oxygen demand and supply resulting in myocyte loss and ventricular failure (7), while DCM is characterized by left ventricular dilation and subsequent contractile dysfunction (8).

Although HF pharmacotherapy has come a long way since diuretics and digitalis were state-of-the-art (9), a long-standing paradigm is that HF with reduced ejection fraction evolves *via* a “final common pathway” (10). Current therapeutic approaches, such as angiotensin-converting enzyme inhibitors (ACEIs) and β-receptor blockers, are relatively etiology agnostic and focus on symptom alleviation (11). This potentially reflects a lack of comprehension of the heterogeneous pathogenic mechanisms in the progression of DCM and ICM.

Dysregulated genes, proteins, and their corresponding biological pathways represent the molecular background of multiple diseases (12). The continuous development of omics technologies and data processing through bioinformatics shed new light on CVD molecular basis (13, 14). Although several standalone transcriptomics and proteomics studies have revealed differentially expressed molecules (DEMs), i.e., genes and proteins, in DCM and ICM, integrated multi-omics analyses of multiple datasets are still sparse.

The present study aims to elucidate the gene expression and proteomic signature of DCM and ICM and explore their molecular characteristics *via* bioinformatics analyses. This approach combines the assessment of mRNA and protein molecules, generating common DEMs, and provides strong evidence for their role in HF. The goal is to identify potential biomarkers and discover novel therapeutic targets by unraveling the complex architecture of HF pathogenesis. Our results accentuate the importance of Extracellular Matrix (*ECM*) organization in HF and stress the need for matrix-based therapies that may attenuate remodeling processes and/or promote cardiac regeneration.

## 2. Materials and methods

### 2.1. Publicly available data collection of DCM and ICM

Transcriptomic and proteomic datasets for DCM and ICM were accessed from the public data repositories GEO (15), ENA (16), and PRIDE Archive (17). The search terms used were “HEART FAILURE” and “HOMO SAPIENS.” Datasets meeting the following three criteria were used: (i) the total number of samples in each dataset should be at least six, incorporating a minimum of three patients and three healthy control samples to ensure statistical significance, (ii) HF patients participating should be strictly diagnosed with either DCM or ICM, and (iii) all samples should be derived from the left ventricle of the heart, as it best reflects the physiological changes of HF (18).

Our search retrieved 6 transcriptomic [GSE3585 (19), GSE57338 (20), GSE5406 (21), GSE116250 (22), GSE133054 (23), PRJEB42485 (24)] and 1 proteomic [PXD008934] (25) dataset for DCM and 7 transcriptomic [GSE76701 (26), GSE57338, GSE5406, GSE46224 (27), GSE116250, GSE48166, PRJEB42485] and 1 proteomic [PXD008934] dataset for ICM. The basic information of all the datasets used is listed in Supplementary Table 1.

### 2.2. Screening for differentially expressed molecules

Gene expression data derived from both microarray and RNASeq methods. Microarray analysis was conducted using the online platform GEO2R. The statistical significance of differentially expressed genes (DEGs) was evaluated through adjusted p-values using the Benjamini and Hochberg (28) procedure and Fold Change (FC) calculations. RNASeq data were quantified, quality controlled, and analyzed using the RaNA-Seq online platform (29). Differential expression analysis was performed using the DESeq2 algorithm (30) and statistically significant results were selected using the adjusted *p*-value (median of ratios). For proteomics data, the analysis report provided in the original paper was utilized. Differentially expressed proteins (DEPs) were determined by utilizing a linear model adjusted for age and sex in the R package limma (31). *P*-values were adjusted for multiple testing using the Benjamini–Hochberg procedure. For all analyses, adjusted *p* < 0.05 and | FC | ≥ 2 were set as the threshold. Above-threshold molecules have dysregulated expression, either upregulated or downregulated, and were considered DEMs in DCM and ICM.

### 2.3. Defining DiSig and IsSig

The intersection of DEMs was demarcated for DCM and ICM independently at first. Molecules that were common in DCM and ICM were identified using a Venn diagram produced by the web tool VENNY (32). The intersecting molecules in DCM were coined as DiSig (Dilated Cardiomyopathy Signature), whereas in ICM as IsSig (Ischemic Cardiomyopathy Signature). Results of this intersection were used in the downstream analyses.

### 2.4. Pathway enrichment analysis

Annotations of cellular components, biological processes, and molecular functions of DiSig and IsSig were determined by Gene Ontology (GO) enrichment analysis, performed using the Metascape platform (33) and PANTHER database (protein analysis through evolutionary relationships) (34) through the OmicsNet platform. Networks of these biological pathways were plotted through Metascape.

### 2.5. Protein-protein interaction (PPI) network analysis and omics visualization

To determine the functional interactions between DEMs, the corresponding protein-protein interaction (PPI) networks were created using the online Search Tool for the Retrieval of Interacting Genes/Proteins (STRING) (35), the online platform Network Analyst (36). Moreover, network integration and visualization of the multi-omics data were achieved by utilizing the online tool OmicsNet (37).

**FIGURE 1:**
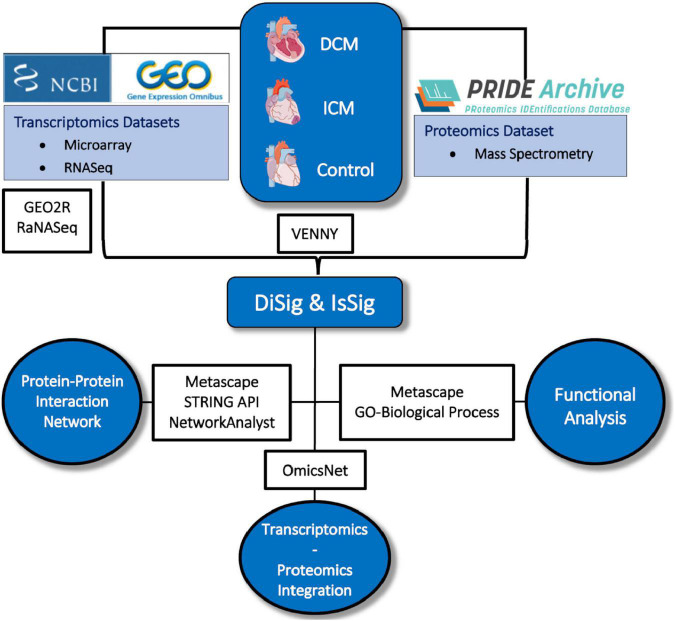
Depiction of the methodology used in this study. Image created using artwork from Servier Medical Art [https://smart.servier.com/].

The entire methodological approach is summarized in Figure 1.

## 3. Results

### 3.1. Identification of DEMs in DCM and ICM samples compared to non-failing samples

Through the microarray DCM dataset analysis, 96 DEGs were identified between the DCM and non-failing groups from GSE3585, of which 66 were upregulated and 30 were downregulated. In GSE57338, 4,319 DEGs were highlighted, of which 4,205 were upregulated and 114 were downregulated. Lastly, a total of 2,450 DEGs were found in GSE5406 (1,377 upregulated and 1,073 downregulated). The analysis of the three RNASeq datasets outlined 210 DEGs from all three datasets, of which 155 were upregulated and 55 were downregulated. Finally, for the proteomic dataset PXD008934, 76 proteins were differentially expressed (54 upregulated and 22 downregulated).

Pertaining to ICM, a total of 82 DEGs were highlighted between the ICM and the non-failing group from GSE76701, of which 39 were upregulated and 43 were downregulated. In GSE57338 a total of 9,095 DEGs were identified between the ICM and the non-failing group, of which 4,357 were upregulated and 4,738 were downregulated, while in GSE5406, 1,599 genes were found to be differentially expressed (610 upregulated and 989 downregulated). The four RNASeq datasets analyzed highlighted a total of 243 DEGs from all four datasets, of which 206 were upregulated and 37 were downregulated. Regarding the proteomic dataset PXD008934, a total of 149 proteins were outlined, 98 of which were upregulated, and 51 downregulated.

The full lists of DEMs mentioned (sorted by *p*-adj) are listed in Supplementary Table 2.

### 3.2. IsSig and DiSig

**FIGURE 2:**
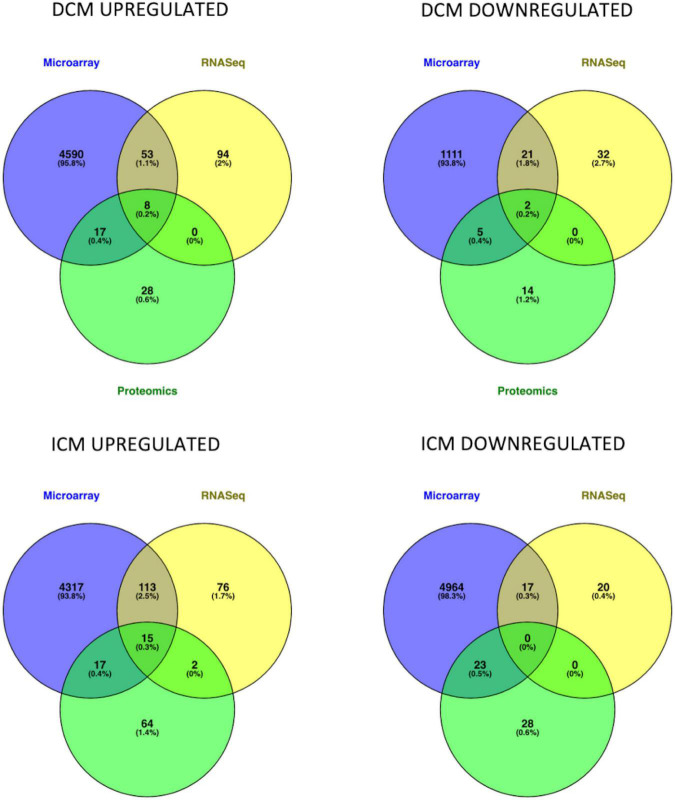
Venn diagrams represent the intersection of genes and proteins in DCM and ICM. The three groups include DEGs derived from microarray (blue), RNASeq (yellow), and proteomics (green) analyses.

Intersection of DEMs deriving from microarray, RNASeq and mass spectrometry dataset analysis showed an overlap in upregulated or downregulated DEMs both in DCM and ICM (Figure 2). List of molecules for each Venn of Figure 2 can be found in Supplementary Table 3.

After intersection of all three (microarray, RNASeq, and mass spectrometry) analyses, eight DEMs were upregulated (*AEBP1*, *CA3*, *HBA2*, *HBB*, *HSPA2*, *SOD3*, *THBS4*, and *UCHL1*) and two were downregulated (*MYH6* and *SERPINA3*) in DiSig. In IsSig, 15 DEMs were upregulated (*AEBP1*, *APOA1*, *BGN*, *CA3*, *CFH*, *COL14A1*, *HBA2*, *HBB*, *HSPA2*, *LTBP2*, *LUM*, *MFAP4*, *SOD3*, *THBS4*, and *UCHL1*) while none downregulated molecule was found. DiSig and IsSig DEMs deriving from this triple intersection are considered valuable indicators of HF.

When intersection of at least two common methods was applied, 78 molecules were upregulated and 28 molecules were down regulated in DiSig, whereas 147 were upregulated and 40 were downregulated in IsSig. These DEMs are used in downstream analyses and results are presented in following sections.

### 3.3. Pathway enrichment analysis

Gene ontology analyses in Metascape were performed on DiSig and IsSig to elucidate the biological functions of DEMs. In enrichment analysis, several biological processes were statistically significant (*p* < 0.0001). Specifically, the detected 78 upregulated DEMs of DiSig regulate extracellular matrix organization, supramolecular fiber organization, cellular response to transforming growth factor-beta stimulus and collagen metabolic process. DiSig downregulated DEMs (*n* = 28) are involved in double-strand break-repair, inflammatory response, mitotic cell cycle, activation of the immune response, regulation of developmental growth, negative regulation of response to external stimulus, and response to wounding.

In IsSig, the upregulated DEMs (*n* = 147) have a role in extracellular matrix organization, ossification, response to transforming growth factor-beta and positive regulation of leucocyte migration. The detected 40 downregulated DEMs are involved in positive regulation of gene silencing by miRNA, cellular response to nitrogen, acute-phase response, protein import into the nucleus, generation of precursor metabolites and energy, phagocytosis and viral process.

The top 100 molecular pathways found are listed in Supplementary Table 4.

**FIGURE 3:**
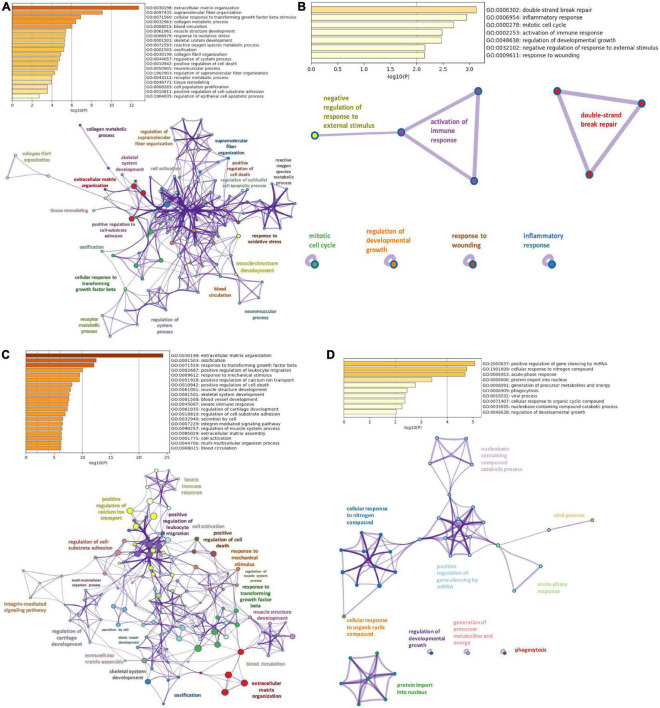
Pathway enrichment analysis. **(A)** Top 20 clusters of biological pathways with the smallest *p*-value, using the upregulated DEMs of DiSig and their corresponding network. **(B)** Top 20 clusters of biological pathways with the smallest *p*-value, using the downregulated DEMs of DiSig and their corresponding network. **(C)** Top 20 clusters of biological pathways with the smallest *p*-value, using the upregulated DEMs of IsSig and their corresponding network. **(D)** Top 20 clusters of biological pathways sorted by *p*-value, using the downregulated DEMs of IsSig and their corresponding network. Each cluster is characterized by a broader general term annotated beside the clusters and encompasses smaller individual terms represented by circle nodes. Their size is proportional to the number of input genes that fall into those terms. The colors represent the clusters’ identities. Terms with a similarity score > 0.3 are linked by an edge (the thickness of the edge represents the similarity score).

Networks of these biological pathways are displayed in Figure 3.

### 3.4. PPI network analysis

Protein-protein interaction networks were constructed using STRING db and NetworkAnalyst online platforms. Two approaches were followed. Firstly, we plotted the networks of DiSig and IsSig using the DEMs that were common in at least two methods as previously described, and secondly, we used the 10 and 15 DEMs of DiSig and IsSig that derived from the intersection of all three analyses to explore their interconnections.

**FIGURE 4:**
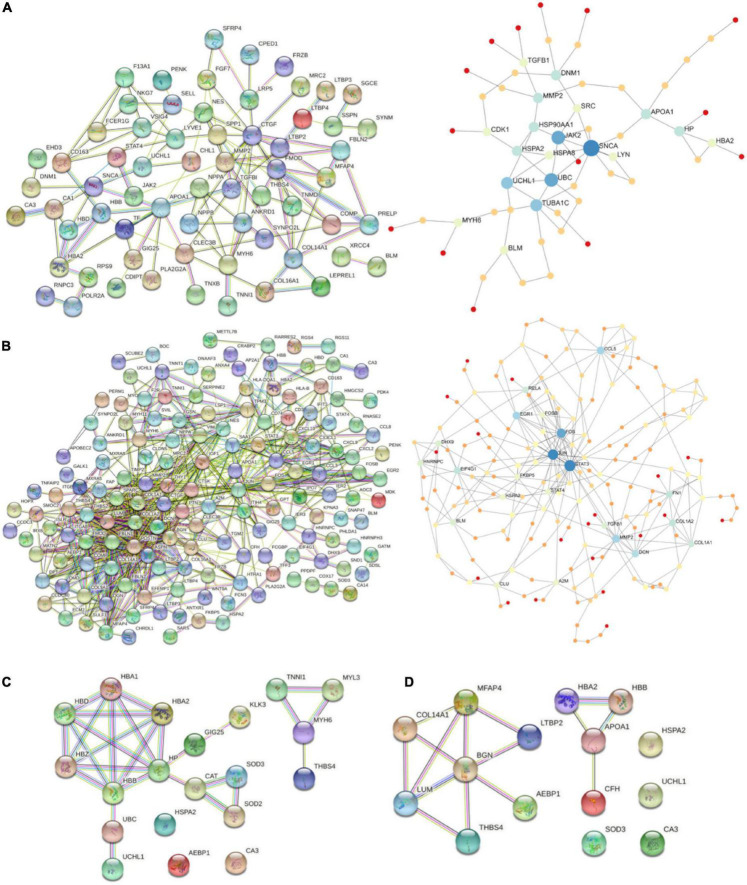
**(A)** PPI-networks of DiSig using STRING db and NetworkAnalyst, respectively. **(B)** PPI-networks of IsSig using STRING db and NetworkAnalyst, respectively. Each node represents a gene, while interacting nodes are linked by edges, the number of which is proportional to their interaction degree. **(C)** PPI networks of the 10 DiSig genes. **(D)** PPI networks of the 15 IsSig genes.

Starting with DiSig, a PPI network analysis was conducted using all 78 upregulated and 28 downregulated DEGs of DiSig. Totally, 122 edges were produced by STRING db analysis (Figure 4A) and the functional network generated by NetworkAnalyst that is depicted in Figure 4B. The STRING interactome database with a high confidence score (900) was selected and the network was trimmed to a minimum. SNCA, UBC, JAK2, TUBA1C, UCHL1, APOA1, MMP2, HSP90AA1, DNM1, and HP were the top 10 nodes according to their network degree value. Similarly, for IsSig the PPI network analysis *via* STRING including all 128 upregulated and 40 downregulated DEGs produced 633 edges (Figure 4C). PPI network analysis for IsSig using NetworkAnalyst generated the following functional network (Figure 4D); STAT3, JUN, FOS, MMP2, CCL5, EGR1, DCN, FN1, COL1A2, EIF4G1 were the top 10 nodes according to their degree value. The PPI Networks of DiSig and IsSig can be found in Supplementary Table 5.

The PPI of the 10 DiSig DEMs was constructed *via* STRING, after Protein Enrichment (Medium confidence = 0.4, first shell interactions = maximum 10 interactors) with the proteins Hemoglobin subunit alpha 2 (HBA2), Hemoglobin subunit beta (HBB), Heat shock-related 70 kDa protein 2 (HSPA2), Myosin-6 Protein (MYH6), Extracellular superoxide dismutase [Cu-Zn] (SOD3), Thrombospondin-4 (THBS4), and Ubiquitin carboxyl-terminal hydrolase isozyme L1 (UCHL1) being interconnected among the 10 DEGs, while the remaining three proteins had no interactions (Figure 4C). The 15 IsSig molecules were analyzed *via* STRING as shown in Figure 4D, and the proteins Adipocyte enhancer-binding protein 1 (AEBP1), Apolipoprotein A-I (APOA1), Biglycan (BNG), Complement factor H (CFH), Collagen alpha-1 (XIV) chain (COL14A1), Hemoglobin subunit alpha 2 (HBA2), Hemoglobin subunit beta (HBB), Lumican (LUM), Latent-transforming growth factor beta-binding protein 2 (LTBP2), Microfibril-associated glycoprotein 4 (MFAP4), and Thrombospondin-4 (THBS4) were found interconnected, while the remaining four proteins had no interactions.

### 3.5. Omics visualization through OmicsNet online platform

**FIGURE 5:**
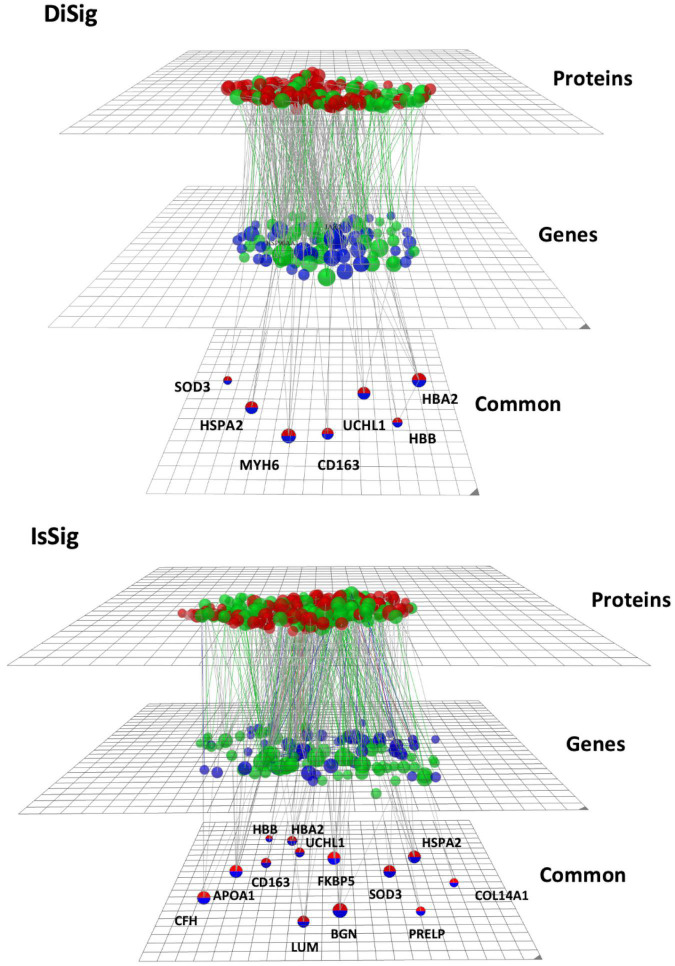
Multi-omics visualization using OmicsNet. The nodes in red represent the proteins, while the nodes in blue represent the genes of DiSig and IsSig, respectively. They are also categorized into three layers: proteins, genes, and common. Green nodes are those associated with the terms extracellular region, extracellular space, and extracellular matrix.

To create and visualize the interconnections and relationships between the genes and proteins derived from transcriptomics and proteomics datasets, the online platform OmicsNet was used. The red nodes represent the protein input, the blue nodes represent the gene input, and the double-colored nodes are the common DEMs (Figure 5).

As previously described, the following pathway analysis in both DiSig and IsSig highlighted the molecular pathways in the Extracellular region, Extracellular space, and Extracellular matrix. These interactions are depicted in Figure 5 with green interconnections. The full list of the molecular pathways by using the PANTHER database is listed in Supplementary Table 6.

### 3.6. Common pathways and genes in dilated and ischemic cardiomyopathy

By comparing the two types of cardiomyopathies leading to HF, both similarities and differences can be deduced. Even though the phenotypic expression is unique in each case, approximately 1/3 (27/100 molecular pathways) of their genomic signature is common when we directly compare the top 100 functional pathways between DiSig and IsSig (Supplementary Table 7). Common biological pathways include extracellular matrix organization, cellular response to stress and transforming growth factor-beta and transmembrane transport of ions. On the contrary, muscle tissue development characterized only DiSig, while immune cell activation and migration were unique in IsSig.

**FIGURE 6:**
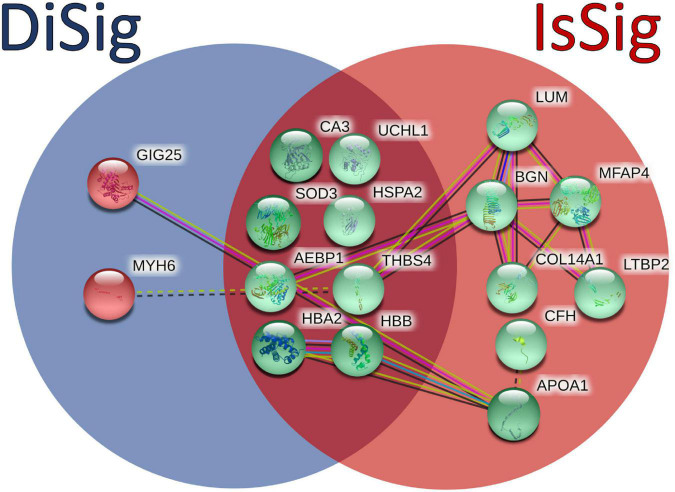
Comparison between the triple cross-validated molecules of DiSig (10 genes) in the blue circle and IsSig (15 genes) in the red circle. Upregulated DEMs are represented in green color, while the downregulated DEMs are represented in red color. The color and type of edges connecting the nodes in this figure (as provided by STRING-db) allocate biological meaning coded as follows: red lines indicate the presence of fusion evidence, green lines of neighborhood evidence, blue lines of co-occurrence evidence, purple lines of experimental evidence, yellow lines of text-mining evidence, light blue lines of database evidence, and black lines of co-expression evidence. In addition, solid lines represent intra-cluster edges and dashed-lines inter-cluster edges.

In addition, a Venn diagram was plotted using the triple “cross validated” DiSig genes (8 upregulated and 2 downregulated) and the IsSig genes (15 upregulated), as shown in Figure 6. The results showed that 8 DEMs were common, while 2 DEMs were unique in DCM and 7 DEGs were unique in ICM.

## 4. Discussion

As shown in the present multi-omics study, a unique molecular signature was deduced for DCM and ICM after intersection of microarray, RNASeq and mass spectrometry analyses with 10 DiSig and 15 IsSig DEMs being the most important finding in our results. In intersection of at least two common methods, DiSig is comprised of 106 DEMs (78 upregulated and 28 downregulated), while IsSig encompasses 187 DEMs (147 upregulated and 40 downregulated). Analyses of pathway enrichment were performed and PPI networks were constructed to detangle the processes and mechanisms of DCM and ICM pathology, using DiSig and IsSig molecules.

Extracellular matrix organization was the most abundantly expressed pathway in both DCM (*p* = 10–12) and ICM (*p* = 10–24), with upregulated genes encoding collagens and metalloproteases. Extracellular matrix (*ECM*) is a well-documented molecular pathway (38) and a major player in cardiac homeostasis. Not only does it provide structural support, but also facilitates force transmission and transduces molecular signals regulating cardiac cell function. In failing hearts, the cardiac interstitium is expanded, by augmentation of both structural and matricellular *ECM* proteins, resulting in alterations in extracellular matrix biochemistry. *ECM* plays a critical role in regulating fibrotic, inflammatory, and even regenerative responses, making it an attractive therapeutic target in HF (39).

*TGF*β signaling pathway is also under investigation for the development of novel therapies for HF. *TGF*β levels are elevated in HF, promoting cardiomyocyte apoptosis and cardiac hypertrophy and playing an important role in heart remodeling (40).

Transmembrane transport of ions is another important pathway found to be upregulated in both DCM and ICM. Ion channels, transporters and pumps comprise only a subset of proteins that are altered during HF with calcium playing a critical role in mediating the cardiac excitation-contraction coupling (41).

By comparing the DEMs of DiSig and IsSig, 8 upregulated molecules were found common. These genes can be categorized as cardioprotective proteins (*HSPA2*, *SOD3*), genes having a major role in remodeling processes (*AEBP1*, *CA3*, *THBS4*, *UCHL1*) and hemoglobins (*HBA2*, *HBB*). *SOD3*, extracellular superoxide dismutase [Cu-Zn], is an antioxidant protein (42), while *HSPA2*, Heat shock-related 70 kDa protein 2, is a molecular chaperone that helps maintain cardiomyocyte protein quality. It can be induced by cellular stress, promoting cell survival, as the toxic to cardiomyocytes’ misfolded proteins, directly contribute to HF (43). Exploration of strategies involving *SOD3* and *HSPA2* may provide therapeutic options against HF and associated systemic inflammation. The carbonic anhydrase enzyme (*CA3*) gene expression is induced in HF due to ventricular stretch inflicted as a consequence of increased ventricular load. Alvarez et al., have presented evidence that elevated *CA3* levels can be used as biomarker for early detection of cardiac hypertrophy and HF (44). The hemoglobin types *HBA2* and *HBB* are overexpressed in DCM and ICM patients, suggesting a potential reciprocal mechanism due to dysregulation in oxygen circulation and general hypoxemia in HF patients.

Thrombospondin-4 (*TSP-4*), a secreted extracellular matrix protein, is involved in myocardial remodeling by regulating the adaptive cardiac responses to pressure overload (45). Another hypertrophic factor, Ubiquitin C-terminal hydrolase L1 (*UCHL1*) is related to fibrosis and has proven to deubiquitinate and stabilize the epidermal growth factor receptor (*EGFR*), promoting cardiac hypertrophy (46). Lastly, Adipocyte enhancer-binding protein 1 (*AEBP1*), a positive regulator of collagen involved in the organization and remodeling of the ECM, was found upregulated in a DCM patients (19).

While IsSig and DiSig share a lot of common elements, IsSig has five additional ECM proteins associated with it (BGN, COL14A1, LUM, LTBP2, MFAP4) and two bloodstream proteins APOA1 and CFH. Collagen type XIV (COL14A1) is major fibrillar collagen produced by fibroblasts and is involved in ECM during the progression of cardiac remodeling in the failing heart (39).

Lumican (*LUM*) is an *ECM* localized proteoglycan associated with inflammatory conditions and known to bind collagens (47). Previous studies in humans and mice indicated that the *LUM* protein levels are increased in cardiac tissues of patients with HF compared to control hearts (48). These findings suggest that *LUM* may contribute to cardiac remodeling, by assisting in fibrinogenesis.

Microfibril-associated glycoprotein 4 (MFAP4) is an ECM protein that is involved in cell adhesion or intercellular interactions. It was demonstrated that MFAP4 deficiency inhibits cardiac fibrosis and ventricular arrhythmias in mice models and therefore may act as a novel therapeutic target for the prevention of cardiac remodeling in HF (49). Latent-transforming growth factor beta-binding protein 2 (LTBP2) is an ECM protein. Bai et al., demonstrated that serum LTBP-2 levels might act as a promising biomarker in HF, as LTBP-2 levels in HF patients are significantly elevated (50). Lastly, Biglycan (BGN) is a protein responsible for muscle development, regeneration, and collagen fibril assembly. In previous studies, it was proven that biglycan is required for the stability of collagen matrix formation, during ECM remodeling (51).

The two downregulated genes specifically in DCM are Myosin Heavy Chain 6 (*MYH6*) and Serpin Family A Member 3 (*SERPINA3*) with its corresponding protein GIG25 (alpha-1-antichymotrypsin). The *MYH6* gene encodes instructions for the cardiac alpha (α)-myosin heavy chain, found in cardiac muscle cells, where it forms type II myosin. Type II myosin generates the mechanical force needed for cardiac muscle contraction in sarcomeres (52). Mutations in *MYH6* may cause a spectrum of cardiac phenotypes associated with contractile dysregulation (53). GIG25, a protease inhibitor, is an acute phase response gene primarily upregulated during inflammation. Tanash et al. concluded that individuals with lower levels of GIG25 protein have a lower risk of developing heart incidents (54). Dysregulation of these two genes/proteins of DiSig can be used to differentiate between the two subphenotypes of HF.

In the work of Kanapeckaitė and Burokienė (55), bulk and single-cell RNA-sequencing and proteomics datasets of the human heart tissue were analyzed. Similar results of tissue remodeling and inflammatory processes were identified as pharmacological targets for DCM and ICM, respectively, despite using a different methodology. Our approaches differ significantly as in our study we accumulated a large number of human samples (in total 252 DCM, 232 ICM, and 221 control heart samples) and achieved gene/protein cross-validation through transcriptomic and proteomic analysis, while their work was based on a mixture of human and murine samples. They also utilized a two-step machine learning pipeline, while we have followed a multi-omic network approach.

Several disease-susceptibility loci of heart failure and cardiomyopathies have been identified in genome wide association studies (56). However, these loci were either not identified as DEGs in our study or they were only differentially expressed in proteomic analysis. It should be acknowledged that genetic variations affecting amino acid coding sequences do not affect the number of final transcripts, mutations leading to protein isoforms cannot be directly linked with transcriptomic or proteomic alterations and, additionally, the number of transcripts does not necessarily correspond to protein abundance since several expression and translation regulators exist in between (57). Further studies are therefore needed to assess the functional significance of genetic alterations, including their transcriptomic and proteomic provoked changes, which can create a predisposition to DCM and ICM.

The results of the multi-omics approach we have integrated propose a total of 17 targets that are potentially of enhanced biological significance as their dysregulation is confirmed on both transcriptomic and proteomic level. These targets can be further investigated as potential therapeutic targets, as a variety of them regulate extracellular matrix (LUM, BGN, MFAP4, COL14A1, LTBP2) and fibrosis (CA3, UCHL1), while others are associated with increased oxidative stress and inflammation in the heart (HSPA2, SOD3). Additionally, the DEMs that were found unique in each disease could serve as biomarkers, by measuring their expression levels in patient samples, leading to patient stratification between the two subtypes of heart failure. To overcome the need of tissue samples for transcriptomic and proteomic analyses, further studies can also identify the best fitted biomarkers in easily accessible biological material, such as blood or plasma.

As with most bioinformatics studies on human diseases, this study has its limitations. Although we detected gene and protein expression in cardiac tissues, as well as several related pathways and mechanisms, these findings need to be confirmed in further studies. Moreover, there are unavoidable differences in samples used such as etiologies and duration of cardiomyopathy, differences in age, gender, and medications, as well as the individual course of the disease, which contribute to the variability of gene and protein expression data. Missing metadata is a common limitation of studies based on public data and heterogeneity on the abovementioned variables is expected. In our study, however, the left ventricle samples used were collected during cardiovascular surgery, suggesting that the disease has already progressed. Subsequently, it can be speculated that the majority of patients were under medication and thus similar confounding factors are expected throughout the whole study population. In addition, true “non-failing” human ventricular tissue is not easy to obtain, as non-transplantable donor hearts are usually exposed to varying degrees of hypoxia which is known to be a potent inducer of BNP gene expression and chemokine (58). Finally, in bioinformatics studies, results can only successfully impute correlation and not causation between differentially expressed genes/proteins and disorders. To assess the causality and functional significance of dysregulated genes in DCM and ICM as for whether these targets contribute to disease pathogenesis or are changes resulting from the disease, different models both *in vivo* and *in vitro* are required; the results of our study can be used for the selection of the molecules further examined in such studies.

An obvious strength of this study is the integration of multiple independent microarray, RNASeq, and proteomic studies accumulating a large number of failing and non-failing hearts allowing for minimizing biases after normalization. To the best of our knowledge, this is the seminal study to cross-validate gene and protein expression as well as differentiate between the two subphenotypes of HF. Additionally, the rather large sample size of our study combined with the strict cutoffs used (*p*adj < 0.05, | FC | > 2) during statistical analysis suggest that the derived results are minimum affected by random variation.

## 5. Conclusion

We aimed to identify the genetic and proteomic signatures of DCM and ICM, using a comprehensive multi-omics analysis. We herein demonstrate that DiSig and IsSig share common gene and protein expression elements, but also exhibit disease-specific molecular pathways. Extracellular matrix dysregulation was highlighted in both DCM and ICM, suggesting an attractive pharmacological target. In total 10 genes/proteins were highlighted in DiSig and 15 genes/proteins in IsSig. Therefore, our findings could provide insights into the pathogenesis of HF and suggest that the uncovered genes can be further investigated as possible novel diagnostic and/or therapeutic agents.

## Data availability statement

The original contributions presented in this study are included in this article/Supplementary material, further inquiries can be directed to the corresponding authors.

## Author contributions

KP: formal analysis, methodology, and writing—original draft. ND: formal analysis, methodology, and writing—review and editing. GR, NA, and GK: writing—review and editing. VM: conceptualization, funding acquisition, writing—review and editing, and final approval. All authors made a significant intellectual contribution and read and approved the manuscript.

## Supplementary material

Supplementary Table 1 The basic characteristics of datasets used, including the number of patients and non-failed controls.

Supplementary Table 2 The lists of DEMs (*p*adj < 0.05, | FC| > 2) in DCM and ICM, respectively, sorted by *p*adj.

Supplementary Table 3 The intersection of DEMs deriving from microarray, RNASeq, and mass spectrometry dataset analysis in DCM and ICM and the list of molecules for each Venn diagram.

Supplementary Table 4 The top 100 molecular pathways found after pathway enrichment analysis on DiSig and IsSig.

Supplementary Table 5 The PPI networks of DiSig and IsSig and their nodes (degree value and betweenness).

Supplementary Table 6 The molecular pathways of DiSig and IsSig by using the PANTHER database.

Supplementary Table 7 Comparison between the top 100 functional pathways of DiSig and IsSig.
