# Supplementary material for: Multi-omics integration to identify the genetic expression and protein signature of dilated and ischemic cardiomyopathy
Source: Front Cardiovasc Med. 2023 Feb 13;10:1115623. doi: 10.3389/fcvm.2023.1115623 (PMC9968758; doi:10.3389/fcvm.2023.1115623)
Supplement: Supplementary Table 1 — The basic characteristics of datasets used, including the number of patients and non-failed controls. [file Table_1.DOCX]

**Basic characteristics of datasets**

DCM, dilated cardiomyopathy; GEO, gene expression omnibus; ICM, ischemic cardiomyopathy; NF, non-failure samples.

| **Microarray datasets** | | | | | | |
| --- | --- | --- | --- | --- | --- | --- |
| **Reference** | **GEO** | **Sample** | **Platform** | **DCM** | **ICM** | **NF** |
| (Barth et al., 2006) | **GSE3585** | Human left ventricular myocardial tissue | Affymetrix Human Genome U133A Array | 7 | - | 5 |
| (Liu et al., 2015) | **GSE57338** | Human left ventricular myocardial tissue | Affymetrix Human Gene 1.1 ST Array | 82 | 95 | 136 |
| (Hannenhalli et al., 2006) | **GSE5406** | Human left ventricular myocardial tissue | Affymetrix Human Genome U133A Array | 108 | 86 | 16 |
| (Kim et al., 2016) | **GSE76701** | Human left ventricular myocardial tissue | Affymetrix Human Genome U133 Plus 2.0 Array | - | 4 | 4 |
| **RNASeq datasets** | | | | | | |
| **Reference** | **GEO** | **Sample** | **Platform** | **DCM** | **ICM** | **NF** |
| (Sweet et al., 2018) | **GSE116250** | Human left ventricular myocardial tissue | Illumina HiSeq 2500 | 37 | 13 | 14 |
| (Ren et al., 2020) | **GSE133054** | Human left ventricular myocardial tissue | Illumina NextSeq 500 | 7 | - | 8 |
| (Darkow et al., 2021) | **PRJEB42485** | Human left ventricular myocardial tissue | Illumina Hiseq 4000 | 5 | 5 | 8 |
| (Yang et al., 2014) | **GSE46224** | Human left ventricular myocardial tissue | Illumina HiSeq 2000 | - | 8 | 8 |
| Wang et al., 2013 | **GSE48166** | Human left ventricular myocardial tissue | Illumina Genome Analyzer II | - | 15 | 15 |
| **Mass Spectrometry dataset** | | | | | | |
| **Reference** | **PRIDE** | **Sample** | **Instrument** | **DCM** | **ICM** | **NF** |
| (Chen et al., 2018) | **PXD008934** | Human left ventricular myocardial tissue | Q Exactive | 6 | 6 | 7 |

Barth, A. S., Kuner, R., Buness, A., Ruschhaupt, M., Merk, S., Zwermann, L., Kääb, S., Kreuzer, E., Steinbeck, G., Mansmann, U., Poustka, A., Nabauer, M., & Sültmann, H. (2006). Identification of a common gene expression signature in dilated cardiomyopathy across independent microarray studies. *J Am Coll Cardiol*, *48*(8), 1610-1617. <https://doi.org/10.1016/j.jacc.2006.07.026>

Chen, C. Y., Caporizzo, M. A., Bedi, K., Vite, A., Bogush, A. I., Robison, P., Heffler, J. G., Salomon, A. K., Kelly, N. A., Babu, A., Morley, M. P., Margulies, K. B., & Prosser, B. L. (2018). Suppression of detyrosinated microtubules improves cardiomyocyte function in human heart failure. *Nature medicine*, *24*(8), 1225-1233. <https://doi.org/10.1038/s41591-018-0046-2>

Darkow, E., Nguyen, T. T., Stolina, M., Kari, F. A., Schmidt, C., Wiedmann, F., Baczkó, I., Kohl, P., Rajamani, S., Ravens, U., & Peyronnet, R. (2021). Small Conductance Ca(2 +)-Activated K(+) (SK) Channel mRNA Expression in Human Atrial and Ventricular Tissue: Comparison Between Donor, Atrial Fibrillation and Heart Failure Tissue. *Frontiers in physiology*, *12*, 650964-650964. <https://doi.org/10.3389/fphys.2021.650964>

Hannenhalli, S., Putt, M. E., Gilmore, J. M., Wang, J., Parmacek, M. S., Epstein, J. A., Morrisey, E. E., Margulies, K. B., & Cappola, T. P. (2006). Transcriptional genomics associates FOX transcription factors with human heart failure. *Circulation*, *114*(12), 1269-1276. <https://doi.org/10.1161/circulationaha.106.632430>

Kim, E. H., Galchev, V. I., Kim, J. Y., Misek, S. A., Stevenson, T. K., Campbell, M. D., Pagani, F. D., Day, S. M., Johnson, T. C., Washburn, J. G., Vikstrom, K. L., Michele, D. E., Misek, D. E., & Westfall, M. V. (2016). Differential protein expression and basal lamina remodeling in human heart failure. *Proteomics Clin Appl*, *10*(5), 585-596. <https://doi.org/10.1002/prca.201500099>

Liu, Y., Morley, M., Brandimarto, J., Hannenhalli, S., Hu, Y., Ashley, E. A., Tang, W. H., Moravec, C. S., Margulies, K. B., Cappola, T. P., & Li, M. (2015). RNA-Seq identifies novel myocardial gene expression signatures of heart failure. *Genomics*, *105*(2), 83-89. <https://doi.org/10.1016/j.ygeno.2014.12.002>

Ren, Z., Yu, P., Li, D., Li, Z., Liao, Y., Wang, Y., Zhou, B., & Wang, L. (2020). Single-Cell Reconstruction of Progression Trajectory Reveals Intervention Principles in Pathological Cardiac Hypertrophy. *Circulation*, *141*(21), 1704-1719. <https://doi.org/10.1161/circulationaha.119.043053>

Sweet, M. E., Cocciolo, A., Slavov, D., Jones, K. L., Sweet, J. R., Graw, S. L., Reece, T. B., Ambardekar, A. V., Bristow, M. R., Mestroni, L., & Taylor, M. R. G. (2018). Transcriptome analysis of human heart failure reveals dysregulated cell adhesion in dilated cardiomyopathy and activated immune pathways in ischemic heart failure. *BMC Genomics*, *19*(1), 812. <https://doi.org/10.1186/s12864-018-5213-9>

Yang, K. C., Yamada, K. A., Patel, A. Y., Topkara, V. K., George, I., Cheema, F. H., Ewald, G. A., Mann, D. L., & Nerbonne, J. M. (2014). Deep RNA sequencing reveals dynamic regulation of myocardial noncoding RNAs in failing human heart and remodeling with mechanical circulatory support. *Circulation*, *129*(9), 1009-1021. <https://doi.org/10.1161/circulationaha.113.003863>
